# Glioma microenvironment-derived CCL2 recruits regulatory T cells and myeloid-derived suppressor cells

**DOI:** 10.1186/2051-1426-3-S2-P72

**Published:** 2015-11-04

**Authors:** Alan L Chang, Jason Miska, Derek A Wainwright, Mahua Dey, Jian Qiao, Peter Pytel, Yu Han, Lingjiao Zhang, Irina V Balyasnikova, Atique U Ahmed, Maciej S Lesniak

**Affiliations:** 1 The University of Chicago, Chicago, IL, USA; 2 Northwestern University, Chicago, IL, USA

## 

Glioblastoma multiforme (GBM) is the most common malignant primary brain tumor in adults with a median survival of 14.6 months despite surgery, radiotherapy, and chemotherapy. One hallmark of GBM is the accumulation of potently immunosuppressive regulatory T cells (Tregs) and myeloid-derived suppressor cells (MDSCs). However, the factors underlying Treg and MDSC trafficking to GBM have not been well established. We identified C-C motif chemokine 2 (CCL2) as a glioma microenvironment-derived chemokine that recruits Tregs and MDSCs that express the cognate receptors CCR4 and CCR2, respectively. First, we found that CCL2 expression is a prognostic factor for GBM patients using data from The Cancer Genome Atlas. High CCL2 expression was correlated with a poor prognosis in both univariate and multivariate Cox regression analyses. We also confirmed the presence of CCL2 in GBM patient samples by immunohistochemical staining. In the syngeneic murine GL261 model of GBM, the major source of CCL2 was macrophages and microglia in the glioma microenvironment rather than tumor cells themselves. Treatment of *ex vivo* isolated microglia and bone marrow (BM)-derived macrophages with GL261 conditioned media resulted in CCL2 production, suggesting that a soluble GL261-derived factor was responsible for the production of CCL2 in microglia and macrophages. To identify potential candidates for this GL261-derived factor, a cytokine array was performed. We found that CCL20 was highly produced by the tumors and was sufficient for the stimulation of CCL2 production by BM-derived macrophages. Implantation of GL261 cells in *Ccl2^-/-^* mice resulted in reduced levels of Tregs and Ly-6C^+^ monocytic MDSCs but not Ly-6G^+^ granulocytic MDSCs. Finally, treatment of mice bearing intracranial GL261 tumors with the small molecule chemokine receptor antagonist C 021 increased median survival ~30%. Thus, the CCL2 chemokine-receptor axis is a potential therapeutic target in GBM as a major mechanism for the recruitment of Tregs and MDSCs.

